# HIV-associated Burkitt lymphoma in a Japanese patient with early submandibular swelling

**DOI:** 10.1186/1756-0500-6-557

**Published:** 2013-12-26

**Authors:** Noriko Komatsu, Yoko Kawase-Koga, Yoshiyuki Mori, Yasuhiko Kamikubo, Mineo Kurokawa, Tsuyoshi Takato

**Affiliations:** 1 Department of Oral and Maxillofacial Surgery, Dentistry and Orthodontics, The University of Tokyo Hospital, 7-3-1 Hongo, Bunkyo-ku, Tokyo 113-8655, Japan; 2 Human Health Science, Faculty of Medicine, Graduate School of Medicine, Kyoto University, 54 Kawaharacho, Syogoin, Sakyu-ku, Kyoto 606-8507, Japan; 3 Department of Hematology and Oncology, Graduate School of Medicine, The University of Tokyo Hospital, 7-3-1 Hongo, Bunkyo-ku, Tokyo 113-8655, Japan

**Keywords:** Burkitt lymphoma, HIV, Submandibular swelling

## Abstract

**Background:**

Patients infected with the human immunodeficiency virus (HIV) are at risk of developing malignancies and have an increased susceptibility to infection. HIV-associated Burkitt lymphoma (BL) is relatively rare in developed countries, but remains prevalent in developing counties and is sometimes compounded by the fact that patients may be unaware that they are HIV-positive.

**Case presentation:**

A 37-year-old Japanese man was referred to our department for diagnosis and management of submandibular swelling. He was unaware that he was HIV-positive at the initial visit. Here, we describe our diagnostic approach, in which we used hematological and immunological investigations, biopsy, fluorescence-activated cell sorting and fluorescence *in situ* hybridization to confirm the diagnosis of HIV-associated BL. The patient has no risk factors for HIV infection, and the source of infection remains unclear.

**Conclusions:**

In this case, submandibular swelling was the first clinical sign of pathology and the patient’s HIV-positive status only became evident later. It is highly likely that BL was triggered by HIV infection.

## Background

Human immunodeficiency virus (HIV) was first reported in 1981, in a cohort of homosexual men with *Pneumocystis carinii* pneumonia [1]. By 2011, the Joint United Nations Program on HIV/AIDS estimated that the number of adults and children living with HIV was between 31.4 million and 35.9 million). In developing countries, especially sub-Saharan Africa, widespread HIV infection has caused severe economic and social problems as a result of decreased life expectancy and increased childhood mortality [2]. HIV-positive patients are more likely to develop malignant disease than healthy individuals because of the immunosuppressive effects of the virus. Moreover, it is not uncommon for patients to be unaware that they are infected with HIV.

Burkitt lymphoma (BL) was first reported in 1958 as a sarcoma of the jaw in a Ugandan patient [3]. Spina *et al*. [4] and Straus [5] have reported that BL accounts for only 1–3% of lymphomas in HIV-seronegative adults, but this figure rises to 15–40% for AIDS-related lymphomas. We report an uncommon case of HIV-associated BL with clinical signs of submandibular swelling in a Japanese man.

## Case presentation

**Figure 1:**
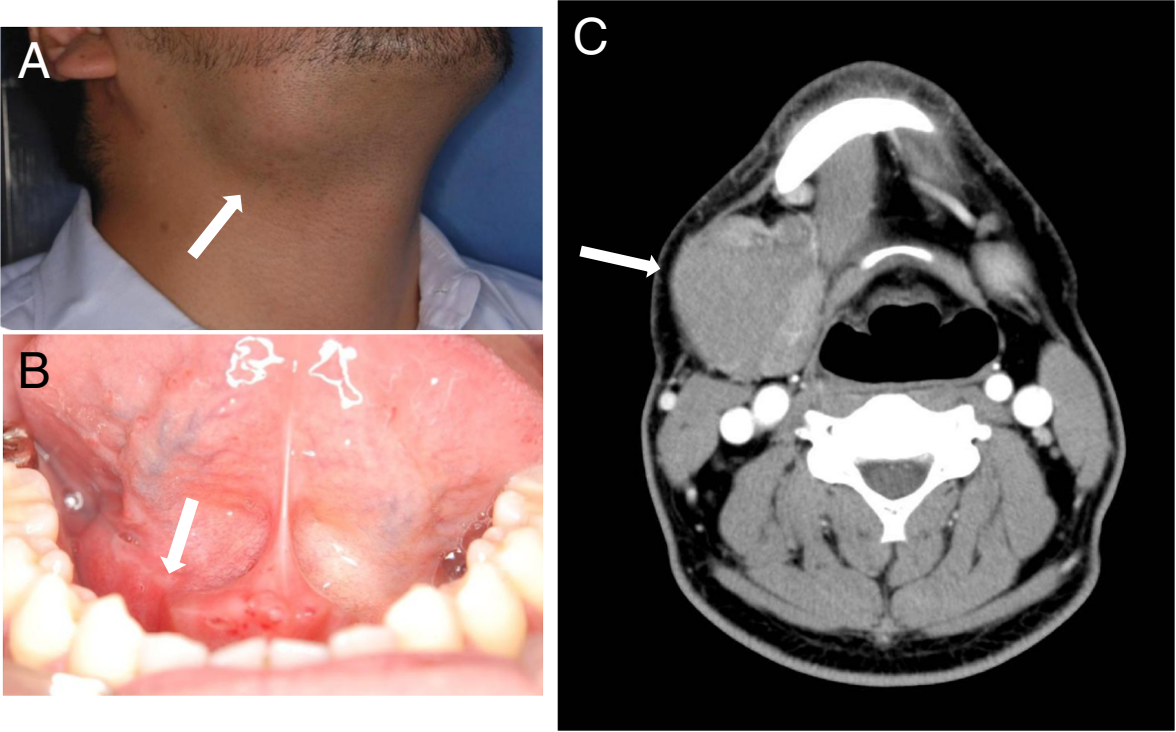
**Clinical and computed tomography findings in the patient. (A)** Right submandibular swelling. **(B)** Swelling in the right side of the floor of the mouth. **(C)** Computed tomography views revealing a cystic lesion involving the right submandibular gland.

**Figure 2:**
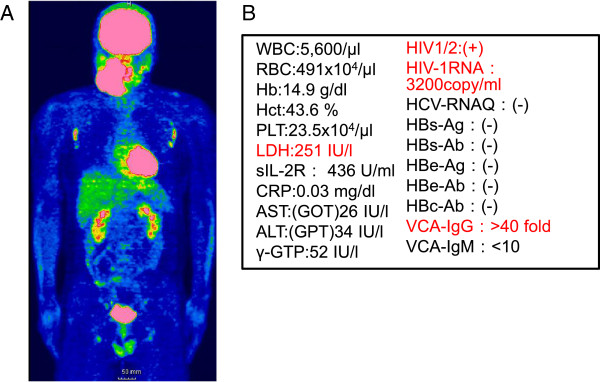
**Flouro-D-glucose positron emission/computed tomography imaging and blood test result. (A)** Fluoro-D-glucose positron emission/computed tomography imaging, showing maximum intensity signals in the submandibular area and lymphadenopathy in the axillary, inguinal and deep cervical nodes. **(B)** Hematological and immunological findings. Red text denotes significantly increased values for these parameters.

A 37-year-old man was referred to the Department of Oral-Maxillofacial Surgery, Dentistry and Orthodontics at the University of Tokyo Hospital for diagnosis and management of a swelling in the right submandibular region, which had been present for approximately 1 month and was associated with sublingual discomfort on eating (Figure 1A). On clinical examination, the swelling was localized to the right side of the floor of the mouth (Figure 1B), and the amount of saliva produced from the right orifice of the right Wharton’s (submandibular) duct was reduced. Computed tomography (CT) views revealed a 50 × 36 mm submandibular adenopathy with a clear border (Figure 1C) but no regional lymph node enlargement. The provisional diagnosis was of submandibular gland inflammation, with a differential diagnosis of malignant lymphoma (ML). Panoramic radiography showed no radiolucent areas that would be characteristic of bony erosion, invasion or destruction, nor were there any radiopaque areas that may be indicative of a sialolith. The medical history was unremarkable. The results of cytology investigations in the right lateral region of the neck indicated a Class II lesion. Exploration of the right orifice of the right Wharton’s duct restored the volume of saliva to normal. Taken together, these findings suggested that the right submandibular swelling was caused by inflammation of the submandibular gland secondary to obstruction of the salivary duct. However, after exploration, the swelling enlarged rapidly and the patient was admitted to hospital for biopsy and further investigations without delay. Fluoro-D-glucose (FDG) positron emission tomography (PET) CT imaging showed maximum intensity signals in the submandibular area and lymphadenopathy in the axillary, inguinal and deep cervical nodes (Figure 2A), findings that are highly suspicious of ML. Surprisingly, immunological and hematological investigations revealed the presence of anti-HIV-1/2 antibodies, HIV-1 ribonucleic acid (3200 copies/ml) and increased LDH levels; however, the levels of sIL-2R and other parameters were normal (Figure 2B). More detailed immunological examination found that anti-VCA-IgG and EBNA antibodies were increased, although anti-VCA-IgM antibodies were at normal levels (Figure 2B). Taken together, these findings indicated a likely diagnosis of HIV-associated malignant lymphoma with no involvement of Epstein Barr virus (EBV). The patient was referred to the Department of Hematology and Oncology at our institution, and, as he was unaware of his HIV-positive status, he was also referred to the Department of Infectious Diseases.

**Figure 3:**
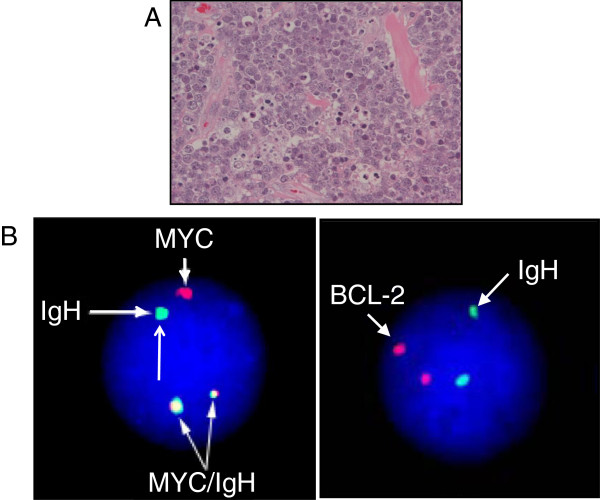
**Histopathological appearance and Fluorescense *in sith *hybridzation analysis. (A)** Histopathological analysis of right submandibular biopsy material, stained with hematoxylin and eosin. This image shows numerous apoptotic cells in a so-called ‘starry-sky’ appearance. **(B)** Fluorescence *in situ* hybridization study showing positive signals for IgH/MYC, but not IgH/BCL-2. Green indicates IgH, red indicates MYC or BCL-2, and yellow denotes areas where green and red signals coincide.

**Figure 4:**
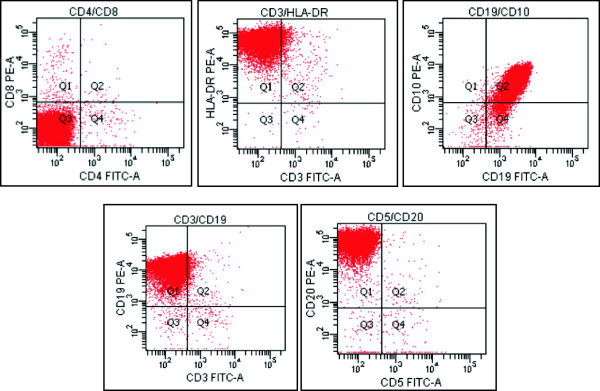
**Representative images from fluorescence**-**activated cell sorting analysis.** The cell population in the malignant tissue was positive for CD10, CD19 and CD20, and negative for CD3, CD4 and CD8.

At the same time, we also undertook a biopsy of the right submandibular area under local anesthesia. The swelling had a clear border covered with a membrane and exhibited no adhesions to the surrounding tissue. Hematoxylin and eosin staining revealed numerous pathognomonic apoptotic cells with a so-called ‘starry-sky’ appearance (Figure 3A). The *c*-*myc* translocation was identified by fluorescence *in situ* hybridization, which identified that the rearrangement was positive for IgH/MYC, but negative for IgH/BCL-2 (Figure 3B). Furthermore, representative fluorescence-activated cell sorting analysis showed negative staining for T-cell associated markers (CD3, CD4 and CD8) and positive staining for B cell-associated antigens (CD19, CD20 and the germinal center-associated marker, CD10; Figure 4). The final diagnosis of HIV-associated BL based on the revised World Health Organization (WHO) classification was confirmed from the clinical, hematological, radiological, histopathological, immunohistochemical and cytochemical analyses. According to a classification based on the Ann Arbor scheme in conjunction with FDG-PET, the disease was diagnosed as being at Stage III.

HIV infection was treated with highly active anti-retroviral therapy at a specialist center, and BL was treated with four cycles of R-HyperCVAD/R-MA chemotherapy administered without radiotherapy. Two years after diagnosis, he continues to receive treatment at the same hospital with no evidence of recurrence. The patient has no apparent risk factors for HIV infection.

## Conclusions

Burkitt lymphoma is a highly aggressive non-Hodgkin lymphoma (NHL) [6]. According to the WHO classification, there are three clinical variants of BL: endemic, sporadic, and immunodeficiency-associated. Endemic BL develops in parts of Africa and New Guinea, in most cases at 4–7 years of age, with boys affected twice as frequently as girls [7]. It can involve the jaw and other facial bones, kidneys, gastrointestinal tract, ovaries, breast, and other extranodal sites. Sporadic BL is a worldwide phenomenon with no specific geographic or climatic association [6], accounting for 1–2% of adult lymphomas and up to 40% of child lymphomas in the United States and Western Europe [7]. Sporadic BL most commonly presents in the abdomen, ovaries, kidneys, omentum, and Waldeyer’s tonsillar rings. Endemic BL is strongly associated with EBV infection, but the etiology of sporadic BL has yet to be defined [8]. Immunodeficiency-associated BL occurs in HIV-infected patients and allograft recipients [9]. This case is highly likely to be an immunodeficiency-associated BL.

HIV-infected patients have a two-fold increased risk of developing malignant disease; in the head and neck the majority of cancers are Kaposi’s sarcoma or oral Kaposi’s sarcoma (68%), with squamous cell carcinoma and NHL accounting for 17% and 13%, respectively, and only 2% diagnosed as BL [10]. Burkitt lymphoma is strongly associated with HIV infection, and HIV-associated BL accounts for approximately 5–40% of cases of HIV-associated NHL [6,8,11]. HIV-associated BL is rare in developed countries, despite being common in developing countries. However, the number of HIV carriers in Japan has recently increased, in part because of internationalization, which may be expected to lead to an increase in HIV-associated BL.

Generally, patients with submandibular swelling visit a dental clinic or are referred to a specialist center for investigation and treatment. In such cases, differential diagnoses that must be considered include inflammation of the submandibular gland, submental ranine, submental lymphadenitis, a tumor of the submandibular gland, a metastatic tumor, and ML. Thus, screening for infectious disease, notably for circulating antibodies against HIV and EBV, is important in cases where suspected BL cannot be differentiated from HIV-associated BL by clinical, radiological, histopathological and immunohistochemical analyses alone. Furthermore, those practicing in oral and maxillofacial medicine should take a history that includes questions regarding sexual history (e.g. sexual intercourse with different partners), travel history (especially to/from countries with many HIV carriers) and history of transfusion of blood products to facilitate differential diagnosis. In this case, the patient is neither homosexual nor has any history of travel to developing countries where HIV is prevalent.

In conclusion, this case emphasizes the importance of an integrated diagnostic approach for the early diagnosis and appropriate treatment of HIV-associated BL in the submandibular region.

## Consent

Written informed consent was obtained from the patient for publication of this case report and any accompanying images.

### Ethics approval

This study was performed in conformity with the Declaration of Helsinki, and was approved by our institutional ethical committee.

## Competing interests

The authors declare that they have no competing interests.

## Authors’ contributions

All authors were involved in the direct diagnosis of the reported patient. All authors were involved in preparation of the manuscript. All authors read and approved the final manuscript.
